# What difference does multiple imputation make in longitudinal modeling of EQ-5D-5L data? Empirical analyses of simulated and observed missing data patterns

**DOI:** 10.1007/s11136-021-03037-3

**Published:** 2021-11-19

**Authors:** Inka Rösel, Lina María Serna-Higuita, Fatima Al Sayah, Maresa Buchholz, Ines Buchholz, Thomas Kohlmann, Peter Martus, You-Shan Feng

**Affiliations:** 1 Institute for Clinical Epidemiology and Applied Biostatistics, Medical University of Tübingen, Silcherstraße 5, 72076 Tübingen, Germany; 2 Medical Clinic, Department of Sports Medicine, University Hospital Tuebingen, Tübingen, Germany; 3 Alberta PROMs and EQ-5D Research and Support Unit (APERSU), School of Public Health, University of Alberta, Alberta, Canada; 4 Institute for Nursing Science and Interprofessional Education, Medical University Greifswald, Greifswald, Germany; 5 Institute for Community Medicine, Medical University Greifswald, Greifswald, Germany

**Keywords:** Imputation, Missing at random, Missing data, EQ-5D, Health-related quality of life

## Abstract

**Purpose:**

Although multiple imputation is the state-of-the-art method for managing missing data, mixed models without multiple imputation may be equally valid for longitudinal data. Additionally, it is not clear whether missing values in multi-item instruments should be imputed at item or score-level. We therefore explored the differences in analyzing the scores of a health-related quality of life questionnaire (EQ-5D-5L) using four approaches in two empirical datasets.

**Methods:**

We used simulated (GR dataset) and observed missingness patterns (ABCD dataset) in EQ-5D-5L scores to investigate the following approaches: approach-1) mixed models using respondents with complete cases, approach-2) mixed models using all available data, approach-3) mixed models after multiple imputation of the EQ-5D-5L scores, and approach-4) mixed models after multiple imputation of EQ-5D 5L items.

**Results:**

Approach-1 yielded the highest estimates of all approaches (ABCD, GR), increasingly overestimating the EQ-5D-5L score with higher percentages of missing data (GR). Approach-4 produced the lowest scores at follow-up evaluations (ABCD, GR). Standard errors (0.006–0.008) and mean squared errors (0.032–0.035) increased with increasing percentages of simulated missing GR data. Approaches 2 and 3 showed similar results (both datasets).

**Conclusion:**

Complete cases analyses overestimated the scores and mixed models after multiple imputation by items yielded the lowest scores. As there was no loss of accuracy, mixed models without multiple imputation, when baseline covariates are complete, might be the most parsimonious choice to deal with missing data. However, multiple imputation may be needed when baseline covariates are missing and/or more than two timepoints are considered.

**Supplementary Information:**

The online version contains supplementary material available at 10.1007/s11136-021-03037-3.

## Introduction

### Background

Patient-reported outcome measures (PROMs) are instruments measuring health from a patient’s perspective. Many are multi-item questionnaires for which raw responses can be converted into composite scores [1]. One of the most widely used health-related quality of life (HRQoL) PROMs is the EQ-5D [2, 3]. Especially in longitudinal studies, PROMs are particularly vulnerable to missing data as respondents may be lost to follow-up, fail to respond, or the responses may be illegible or implausible [1, 2, 4–7]. Unit non-response (UNR) occurs when all items of a scale are missing. Item non-response (INR) occurs when only certain items of a scale are missing [1, 8, 9]. Both non-response types can affect the calculation of the composite score [1] and may result in a loss of statistical power and introduce bias, depending on the quantity of missing values [10, 11].

Recommendations on dealing with missing data [1, 2, 4, 5] depend on the missingness mechanisms, which are categorized according to the relationship between missing values and their dependence on observed and unobserved variables [4, 11]: missing values are missing completely at random (MCAR) when they are independent of both observed and unobserved data. Missing at random (MAR) occurs when missingness is systematically related to observed data but not to unobserved data, and missing not at random (MNAR) occurs when missingness depends on unobserved data [4, 8, 12]. Excluding respondents with missing data from the analysis (complete cases: CC) is rarely adequate and only unbiased if data are MCAR [6, 8, 13]. Multiple imputation (MI) usually produces less biased estimates than several other missing data approaches such as last observation carried forward (LOCF) or mean imputation [2, 4, 8, 11, 14]. As a consequence, MI is currently a widely recognized tool for dealing with missing data [14]. Although MI is the state-of-the-art method for dealing with missingness [2, 4, 8, 14], it has also been argued that longitudinal regression techniques such as mixed models (MMs) can be used regardless of the presence of missing data [4, 14, 15] providing similar results to MI [16, 17]. Estimates are consistent in the MAR case if the predictors of the missing status are also included as covariates in the MM [11].

Another issue regarding missing values is that many PROMs scales, including the EQ-5D, cannot be scored if at least one item is missing [1]. Research concerning multi-item instruments focusses on performance assessments of MI at item- or score-level. Although it is argued that imputation at item-level may yield additional information and therefore improve the accuracy of the imputation [1], this question has not been adequately resolved in the current literature, as evidence has been heavily dependent on the given sample size, missingness proportions and patterns [1, 2].

The developers of the EQ-5D do not provide conclusive guidelines on how to handle missing EQ-5D data. Whether MI is necessary in combination with longitudinal MM analyses is currently debated [4] and the question of the best MI approach (by item or by score) is not yet settled. This paper aimed to elaborate findings on the general necessity of MI in EQ-5D panel data analyses and the performance of MI at item or composite score-level and will thus provide EQ-5D users with clearer recommendations on how to appropriately account for missing data in statistical analyses. Comparisons of these four approaches were performed by analyzing data from two longitudinal studies, using the observed missing data pattern of one dataset, and simulating missing data patterns for the second dataset.

## Materials and methods

### Instrument

#### EuroQol five-dimension (EQ-5D)

The EuroQol five-dimension (EQ-5D) is a generic HRQoL instrument which is self-administered and available in numerous language versions. The EQ-5D consists of two parts: the 20 cm visual analog scale (EQ-5D VAS) and the EQ-5D self-classifier, which captures five dimensions of HRQoL, each represented by one item: mobility, self-care, usual activities, pain/discomfort and anxiety/depression [18]. The most recent version of the EQ-5D uses a five-level response option (EQ-5D-5L) corresponding to the severity of health impairments (*1* = *no problems, 2* = *slight problems, 3* = *moderate problems, 4* = *severe problems, 5* = *extreme problems*) in each of the five dimensions and resulting in 3,125 (= 5^5^) possible health states. These health states can be converted into an overall index score using population/country-specific weights. Index score ranges differ across weights with higher values representing better health.

### Data sources

#### Dataset 1: Canadian cohort study (ABCD)

We explored the EQ-5D-5L responses of a prospective two-year Canadian cohort study, which included patients with type-II diabetes (Alberta's Caring for Diabetes (ABCD); n = 2,040) [19]. Patient outcomes were assessed at three timepoints (baseline, 1 year, 2 years). The EQ-5D-5L index was derived using the Canadian value set [20]. The EQ-VAS was not included in the ABCD dataset. The covariates included in analyses were age, gender, a single self-rated health item (SHR), marital and academic status.

#### Dataset 2: German rehabilitation multi-center study (GR)

We also analyzed a German multi-center study that included inpatient populations of orthopedic and psychosomatic rehabilitation patients with baseline (T0) and post-treatment (T1) assessments (German Rehabilitation (GR); n = 691) [21]. The same covariates used for the ABCD were available in the GR dataset and included in analyses. The EQ-5D-5L index scores were calculated using the German 5L value set [22].

### Design and procedure

**Fig. 1:**
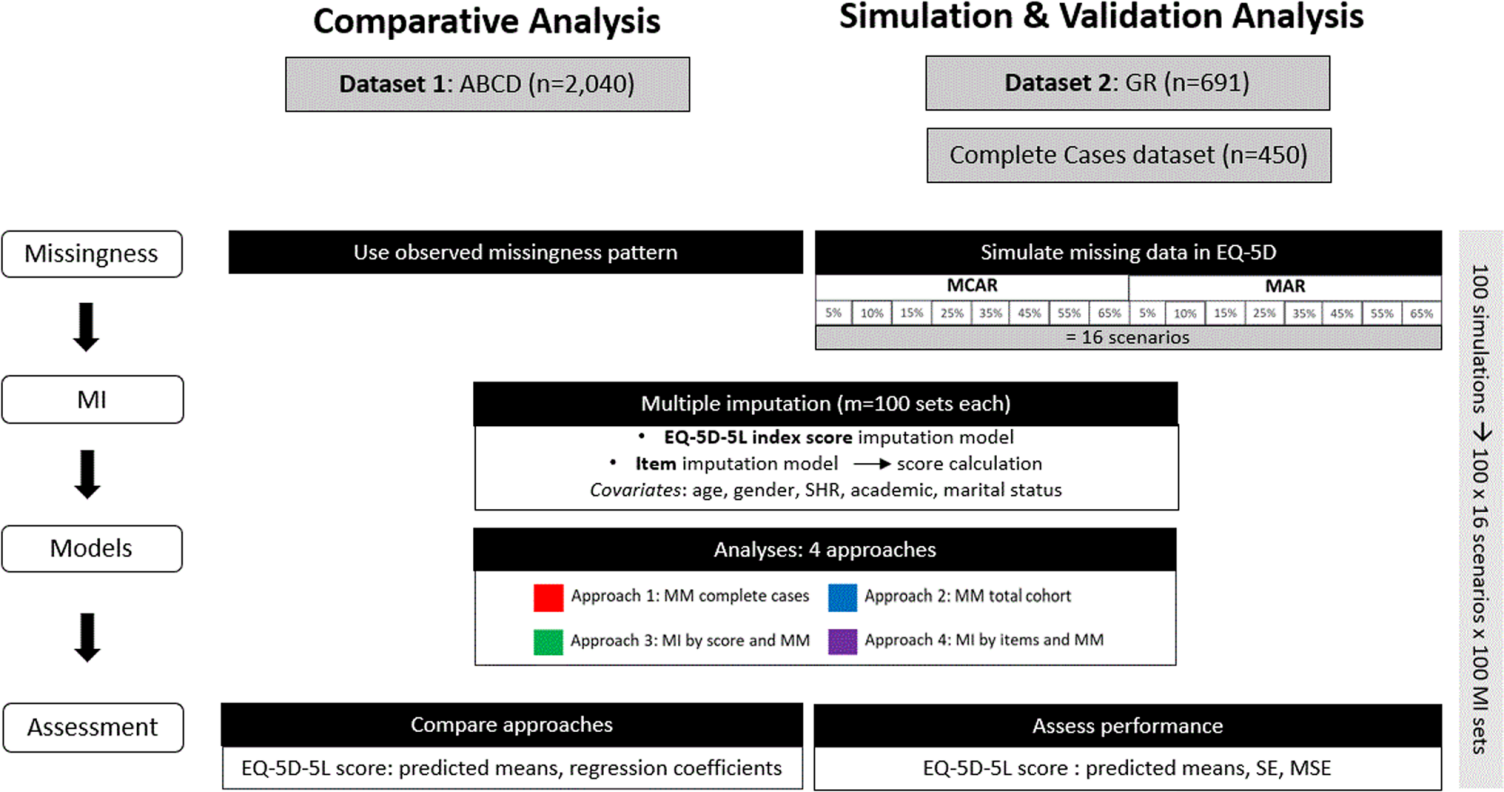
Study procedure. *MCAR* missing completely at random, *MAR* missing at random, *MI* multiple imputation, *MM* mixed model, *SE* standard error, *MSE* mean squared error, *SHR* single self-rated health item

To fully assess how different approaches to handling missing EQ-5D data compare, we conducted both an empirical analysis on observed missingness patterns (Dataset 1: ABCD) as well as a simulation analysis for which we controlled the missingness patterns (Dataset 2: GR). Figure 1 provides an overview of the study procedure.

### Missing data simulation (GR dataset)

**Fig. 2:**
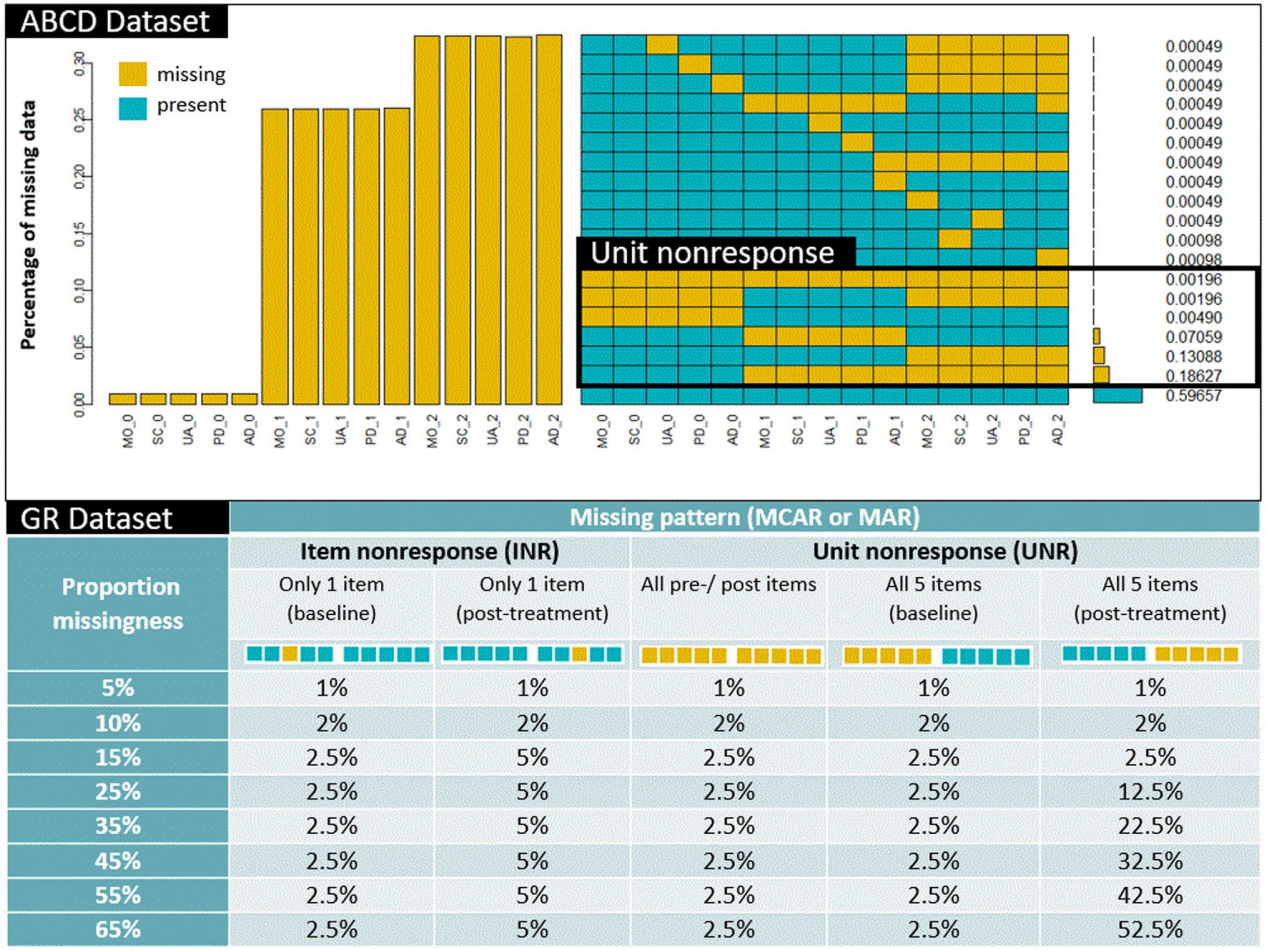
Original patterns of missing EQ-5D-5L data in the ABCD dataset and missing data patterns simulated in the GR dataset (target percentages of missing data). *MO* mobility, *SC* self-care, *UA* usual activities, *PD* pain/discomfort and *AD* anxiety/depression, 0: baseline evaluation, 1: evaluation at 1 year and 2: evaluation at 2 years

We simulated MAR (GR-MAR) data at overall levels of 5%, 10%, 15%, 25%, 35%, 45%, 55% and 65% of missing (8 missing data patterns). The predominant missing data pattern commonly seen in EQ-5D data is UNR [2] (see also **Fig. ****2**, ABCD dataset). To ensure a realistic simulation of missingness, we mimicked these patterns for the GR dataset: INR did not exceed 7.5%, whereas UNR had a maximum of 57.5% (in the case of 65% overall missingness). UNR was at most 5% at T0 with increasing missingness proportions at T1 according to the overall missingness percentage (**Fig. ****2**). In all simulations, missing values were solely generated for the EQ-5D-5L and not for other covariates. The complete simulation process was repeated 100 times to obtain stable estimates of the performance measures.

MAR datasets were generated according to the multivariate amputation procedure by Schouten et al. using the function *ampute* [23] from the R-package mice [24]. We assumed that the probability of the data being missing was related to age, gender, marital status, academic level and a single self-rated health (SRH) item (auxiliary variables) [25]. Using these auxiliary variables, weighted sum scores were generated to determine the probability of a case of being missing for a given variable [26, 27]. We specified the weights by looking at the percentage of missing values in the ABCD dataset for each level of categorical auxiliary variables or the correlation between numerical auxiliary variables and the percentage of missingness, respectively (e.g., age was negatively correlated with the drop-out rate, so a negative sign was assigned to the weight of age). In a last step, based on the weighted sum score, each case received a probability of being missing for a given variable. For the allocation of these probabilities, we applied a right-tailed logistic distribution function, so that cases with higher weighted sum scores have a higher probability of being missing than cases with lower weighted sum scores [27].

### Analysis approaches

The following four analysis approaches of handling missing data were applied to both the ABCD and the simulated GR datasets: 1) MM using complete cases (CC), which included only patients who completed the EQ-5D-5L questionnaire at both timepoints; 2) MM using all available data without MI, which included all available subjects, even if the outcome variable is partially missing at certain timepoints; 3) MM after MI of the EQ-5D-5L index scores; and 4) MM after MI of EQ-5D-5L items.

### Multiple imputation (GR and ABCD dataset, approaches 3 and 4)

All imputation models were performed using MI by expectation–maximization (EM) algorithm on multiple bootstrapped samples of the original incomplete data to draw values of the complete-data parameters, using the *Amelia* package (R Software) [2, 14, 28–32]. Since the outcome variable was recorded over time, an imputation model for trends in time by smooth basis functions of the time variable was used [32, 33]. Variables in the MI models included the outcome (EQ-5D-5L), as well as the covariates incorporated in the MMs and used for the simulation of MAR data (age, gender, a single self-rated health, marital and academic status). MI at the index score-level was conducted after EQ-5D scores had been calculated from available data and the MI model included both EQ-5D scores as well as non-missing items. In case of MI at item-level, MI was conducted using all available EQ-5D items, after which the EQ-5D scores were calculated with the respective value set. A set of 100 imputations was implemented for each dataset at index and item levels. Estimates were pooled according to Rubin's rules [4, 28, 34].

### Mixed models

Using MMs, the changes of the EQ-5D-5L score over time were estimated, with timepoints being nested within the participants. For all four approaches the MMs were specified with fixed effects for time, age, sex, academic status, single self-rated health, and a random intercept for subjects. MMs only incorporated the EQ-5D on score-level, so whenever an item was missing, a score could not be calculated and did not provide additional information in modeling the EQ-5D score across time in approaches 1 and 2. For approaches 3 and 4 all analyses were carried out on the 100 imputed datasets (ABCD) for each simulation set (GR datasets) (= 8 missingness proportions × 100 imputation sets × 100 simulations).

### Comparison of the approaches and performance assessment

For each simulation a predicted mean EQ-5D-5L index score and standard errors (SE) were obtained for the ABCD and the GR datasets. For the approaches with MI, Rubin-adjusted standard errors across the imputed datasets were calculated. For the GR dataset, the mean squared error (MSE) between the estimated EQ-5D-5L and the actual EQ-5D-5L index was additionally calculated to assess performance of the models. Estimated means and the corresponding SE and MSE were averaged across the 100 simulations. All MI and MM analyses were performed in R (version 3.6.3).

## Results

### Respondent characteristics in the two datasets

#### Dataset 1: Canadian Cohort (ABCD)

The ABCD’s EQ-5D-5L instrument completion rate (scale level) at baseline was 99.0% (*n* = 2019), after one and two years 73.8% (*n* = 1507) and 67.4% (*n* = 1374), respectively. Table 1 shows the baseline characteristics of the ABCD dataset. 96.4% (*n* = 1967) of the participants provided complete baseline covariate information. Covariates with missing data included age (2.7%), gender (0.6%), marital status (1.4%), academic status (0.6%) and single self-rated health (1.8%). Figure 2 displays the pattern of missing values in the ABCD dataset: the majority of the samples (59.7%) had complete EQ-5D-5L values in all three evaluations. In total, 99.3% of all missing patterns were UNR patterns. Respondents with complete cases had better EQ-5D-5L index at baseline (mean = 0.809) than patients who were missing data (mean = 0.774). In addition, respondents with missing data had significantly poorer SRH (*p* < 0.001), lower academic achievement (*p* < 0.001) and different marital status (*p* = 0.04) (Supplement Table 1), suggesting that the data are unlikely to be MCAR. The EQ-5D-5L index score values ranged of -0.148 (worst health) to 0.949 (best health). The descriptive mean values of the EQ-5D-5L index scores deteriorated from baseline over the two follow-up years (Table 1) describing an overall progression of the diabetes type-II disease in the cohort.

**Table 1:** Baseline characteristics of the ABCD (*n* = 2040) and GR dataset (*n* = 450)

	ABCD dataset (*n* = 2,040)		GR dataset (CC) (*n* = 450)	
	*n*	Mean (SD) or *n* (%)	*n*	Mean (SD) or *n* (%)
Age mean (SD)	1985	63.07 (13.41)	450	53.13 (10.43)
Gender Male *n*(%) Female *n* (%)	2027	1,110 (54.8%) 917 (45.2%)	450	150 (33.3%) 300 (67.7%)
Marital status Never married *n* (%) Now married or common law *n*(%) Separated or divorced *n*(%) Widowed *n*(%)	2012	1,459 (72.5%) 127 (6.3%) 230 (11.4%) 196 (9.7%)	450	59 (13.1%) 305 (67.8%) 64 (14.2%) 22 (4.9%)
Academic status No formal schooling *n*(%) Completed grade school *n* (%) High school *n* (%) College/University *n* (%)	2028	11 (0.5%) 265 (13.1%) 813 (40.1%) 939 (46.3%)	450	68 (15.1%) 244 (54.2%) 112 (24.9%) 26 (5.8%)
Single self-rated health (SRH) Excellent Very good Good Fair Poor	2004	71 (3.5%) 611 (30.5%) 926 (46.2%) 331 (16.5%) 65 (3.2%)	450	0 (0%) 27 (6.0%) 187 (41.6%) 200 (44.4%) 36 (8.0%)
EQ-VAS baseline evaluation		No data	450	58.85 (19.15)
EQ-5D-5L Index score				
EQ-5D-5L index baseline mean (SD)	2019	0.795 (0.169)	450	0.706 (0.241)
EQ-5D-5L index second evaluation mean (SD)	1507	0.793 (0.168)	450	0.762 (0.235)
EQ-5D-5L index third evaluation mean (SD)	1374	0.788 (0.173)		Not applicable

*CC* complete cases

#### Dataset 2: German Cohort (GR)

Descriptive statistics of the baseline characteristics for the 450 patients included in the complete case cohort are shown in Table 1. The mean age was 53.13 years (SD = 10.4) with a proportion of 67.7% (*n* = 300) females. The EQ-5D-5L index score values at the first evaluation ranged from − 0.458 to 1, and from − 0.197 to 1 at the second evaluation. The mean EQ-5D-5L index score was estimated to be 0.706 (SD = 0.241) at T0 and 0.762 (SD = 0.235) at T1, indicating improvement in the quality of life. This improvement was opposed to the results in the Canadian cohort due to the interventional nature of the GR study. Supplementary Fig. 1 shows the pattern of the simulated missing values in the GR dataset.

### Analysis of the approaches

#### Dataset 1: Canadian Cohort (ABCD)

**Fig. 3:**
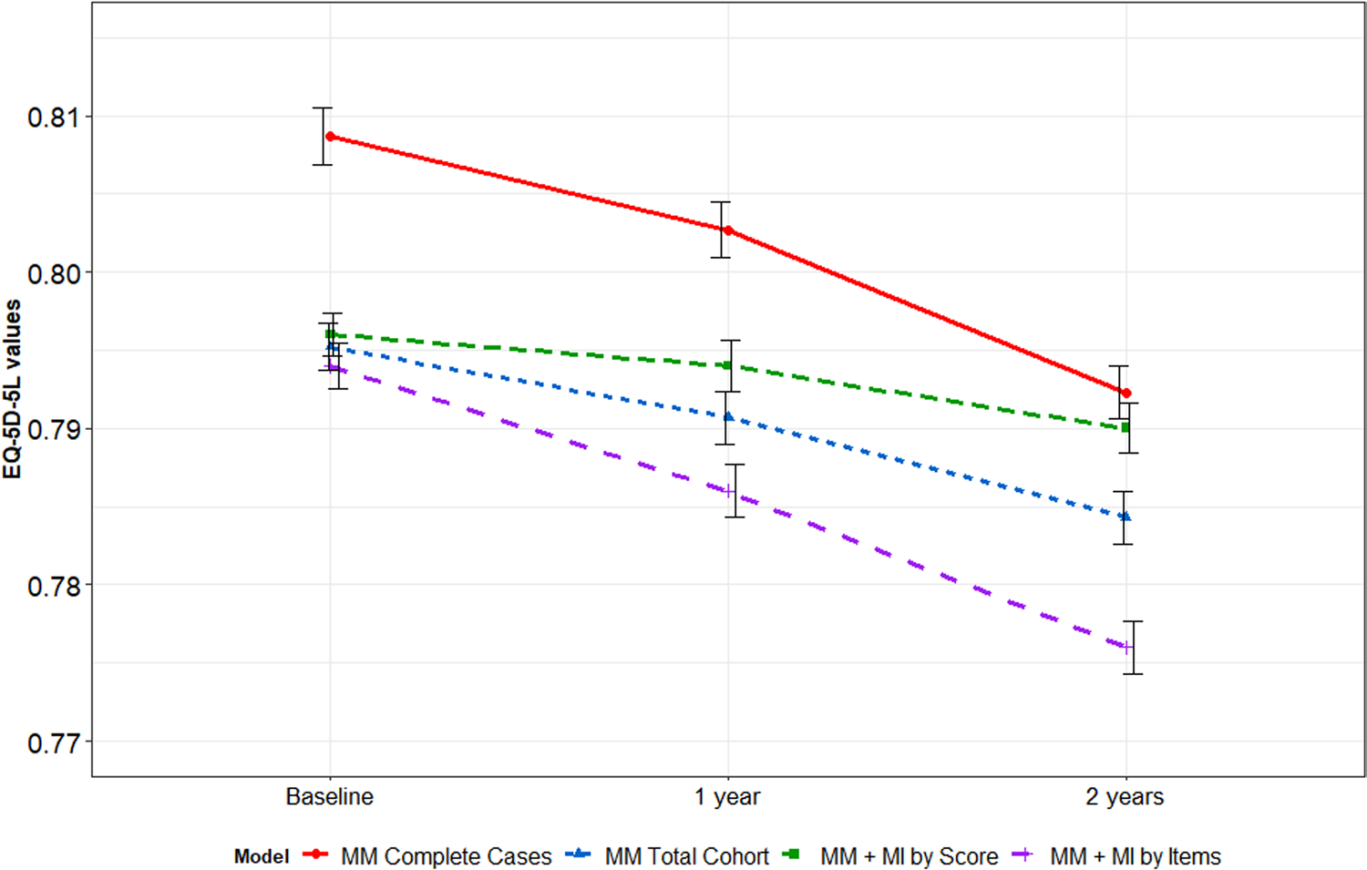
Predicted mean EQ-5D-5L (*ABCD dataset*) over time according to the different approaches

Figure 3 and Supplement Table 2 show the estimated mean EQ-5D-5L index score over time for the ABCD dataset according to the different approaches. CC data analysis (approach-1) resulted in the highest mean index scores at all-timepoints (visit-1 = 0.809, visit-2 = 0.803, visit-3 = 0.793). Approach-2, 3 and 4 estimated similar baseline mean scores (0.794–0.796); however, approach-4 (MM after MI by items) produced the lowest scores at follow-up evaluations (visit-2 = 0.786, visit-3 = 0.776) (Fig. 3). The largest slope of change was observed for approach-4 (*β*_time_: − 0.008), while the smallest slope was observed for approach-3 and 1 (*β*_time_: − 0.002) (Fig. 3).

**Table 2:**
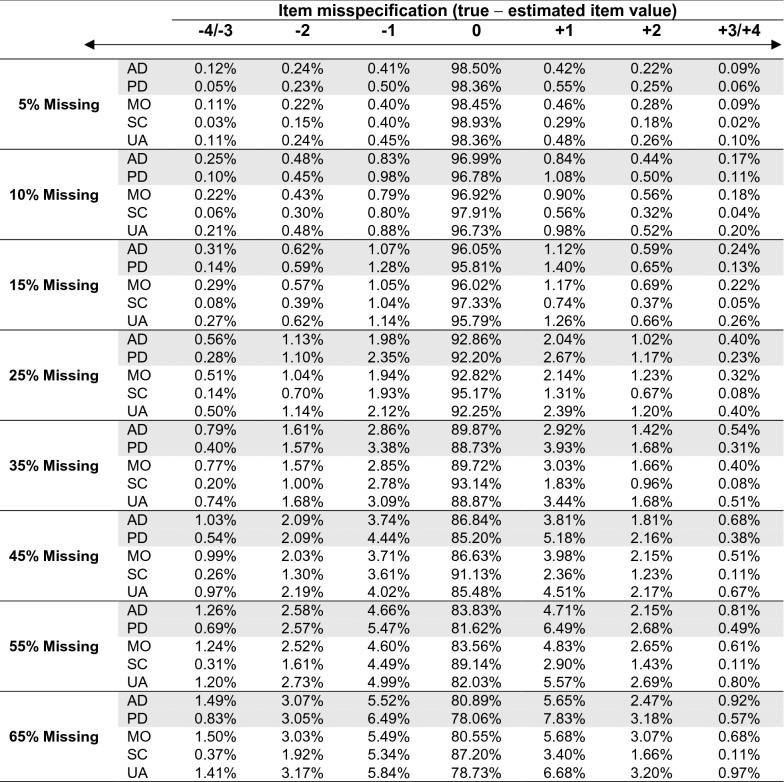
Percentage of item misspecifications after multiple imputation by items (GR dataset)

*Gray shaded* symptoms item, non-shaded items: functions items, *AD* anxiety/depression, *PD* pain/discomfort, *MO* mobility, *SC* self-care, *UA* usual activities

Here we display the percentage of misspecifications of item levels after multiple imputation by item. This was done by calculating the difference between true value from the GR CC dataset and the imputed estimate. Percentages were presented over all imputation and simulation sets

#### Dataset 2: German Cohort (GR)

**Fig. 4:**
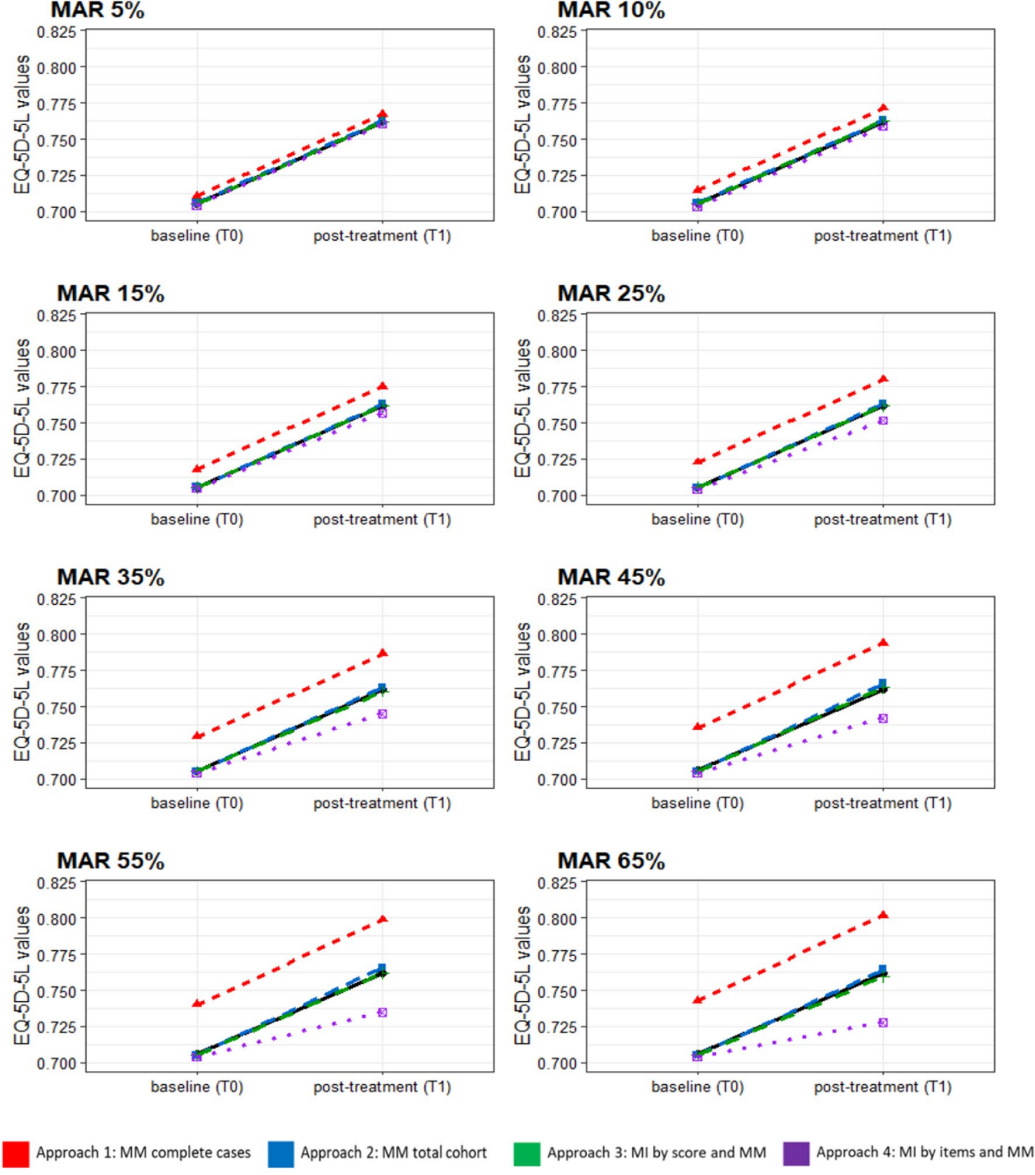
Predicted mean EQ-5D-5L index scores (*GR dataset*) over time according to the different approaches; MAR scenario. *MAR* missing at random, *MM* mixed model, *MI* multiple imputation. The black lines represent the true scores

Figure 4 and Supplement Table 3 show the results of the predicted mean values of the EQ-5D-5L index score over time for the MAR missing data patterns. In line with the results of the ABCD data, approach-1 yielded the highest estimates of all approaches, increasingly overestimating the score at both evaluation timepoints with higher percentages of missing data. Also consistent with the results in the ABCD data, approach-4 produced fairly precise predictions for T0, but progressively underestimated the score at T1 with increasing amount of missing data with a decreasing slope of change from baseline. The estimated beta coefficient for time was very inaccurate at larger missing percentages compared to the true β_time_ from the MM on the full dataset (β_time_GR_ = 0.026), even changing sign (*β*_time_55%_ = -0.003, *β*_time_65%_ = -0.010) (see Supplementary Table 4). Approach-2 and approach-3 were similarly close to the observed EQ-5D-5L index scores of the GR complete case dataset for all percentages of missingness.

**Fig. 5:**
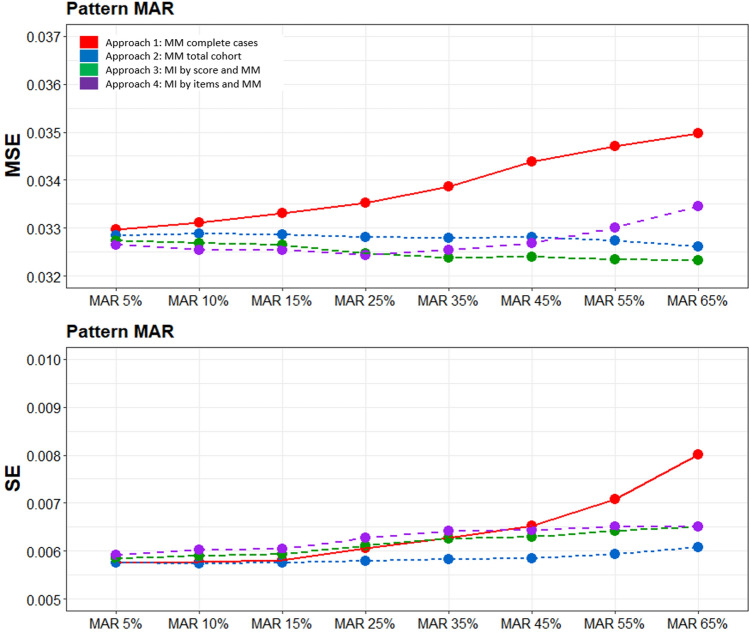
Mean square error (MSE) of the models and standard error (SE) of predicted values (post-treatment T1) using different percentage of missing data (*GR dataset*); MAR scenario. *MAR* missing at random, *MM* mixed model, *MI* multiple imputation, *MSE* mean squared error, *SE* standard error

Figure 5 presents the MSE and SE across the simulated missing data patterns with different percentages of missing values. For the MAR datasets, approach-1 provided the most inaccurate predictions yielding the largest MSE compared to all other approaches with an increasing MSE across the increasing proportions of simulated missing data. The MSEs obtained from approach-2 and approach-3 exhibited only minor differences, the performance of approach-4 started to deteriorate after 45% of missing data. With respect to the SE of the estimated means, approach-1 showed the highest SE values for missing percentages larger than 45%. The SEs for the other approaches were similar, with approach-2 producing the smallest SE across all missing data proportions, however with slightly larger MSEs than approach-3.

To give further insight into the imputation precision at item-level, Table 2 shows the percentage of correctly specified and misspecified item levels under MI by item. The differences between true item values from the GR CC dataset and the estimated item levels for the datasets with simulated missingness were calculated (true value—estimated value) and categorized into correct specification and degree of over- or underestimations. As expected, item levels were increasingly misspecified at higher percentages of missingness and strong over- or underestimations (− 3/− 4 or + 3/ + 4) were less common than small misspecifications (− 1 or + 1).

## Discussion

Although the EQ-5D-5L is an extensively used health outcome instrument, no guidance is currently provided in the user manual on how to deal with missing values [35]. We applied four different approaches of modeling the outcomes of the EQ-5D-5L using a dataset with observed missingness (ABCD dataset) and datasets with simulated missingness (GR datasets) to assess whether MI is necessary before performing MM and to evaluate if imputation at score- or item-level is preferable.

These analyses demonstrated that MM without MI and MM after MI by score yielded very similar estimates (ABCD and GR datasets) with approximately unbiased coefficients (GR datasets). In contrast, the CC approach consistently produced the highest EQ-5D-5L scores (ABCD and GR datasets) overestimating the true EQ-5D-5L scores with increasingly larger MSEs and SEs at higher percentages of missing data (GR datasets). MM after MI at item-level produced the lowest EQ-5D-5L estimates (ABCD and GR datasets), particularly at follow-up timepoints with a markedly deteriorating performance at ≥ 35% of missing data (GR datasets). However, with respect to the scale of the EQ-5D-5L, no dramatic differences in the EQ-5D-5L estimates could be found across all four approaches (Figs. 3 and 4).

MI is commonly advocated as the most appropriate technique for addressing missing data in a variety of circumstances [11, 13, 31]. However, several simulations studies have shown that MM without MI produce similar results if data are either MCAR or MAR [4, 25]. The observed overestimation of the EQ-5D-5L scores, especially with higher percentages of missingness proportions in the simulation sets, can partially be explained by the fact that patients with a worse general health status are more prone to be lost to follow-up and the simulated MAR missingness mechanism [36, 37].

Our simulation analysis was performed only for EQ-5D-5L outcomes, whereas covariates were assumed to be complete. Under this premise, MM with all available data without MI and MM after MI by score were found to yield similar results with approximately unbiased coefficients even at higher percentages of missingness for MAR scenarios. This is in accordance with Twisk and colleagues who suggested that there is no obvious gain from handling missing data using MI before performing a MM analysis on longitudinal data [4]. As MM analysis without MI is clearly computationally more efficient, it is the most parsimonious choice [4, 38, 39].

On the other hand, although MM techniques do not exclude respondents with missing values, cases with incomplete observation of any covariate will still be excluded. MM requires that the model involves all the variables needed to make the MAR assumption valid, and will hold only if the outcome has missing values, not if the baseline covariates have missing values [40]. Our results showed that in contrast to the GR dataset with no missing baseline covariates, MM without MI and MM after MI by score in the ABCD dataset with observed covariate missingness did yield slightly different coefficients and slopes. The differences were small as the ABCD dataset had low baseline covariate missingness. These differences could be more striking if more data were missing at baseline, for which MI potentially hold an advantage over MM only [4]. Additionally, when auxiliary variables are associated with drop-out, they can be included in predicting the missing data in the MI model without being included in the MM analysis, which may increase efficiency [16, 41]. We therefore suggest basing the decision on whether to apply MI before MM on the magnitude of covariate missingness. If covariate missingness is low, MM without MI seems to be the most reasonable approach. However, further in-depth research is needed to understand this phenomenon.

When implementing MI for multi-item instruments, there is also little guidance on whether imputation should be applied at score or item-level [1]. The comparison of our approaches revealed that MM after MI by score provided more reliable estimates than MM after MI by items (GR datasets), particularly at proportions of 35% missingness and higher. In the GR as well as in the ABCD dataset the estimated EQ-5D-5L scores at follow-up were consistently lower for MM after MI by items than for MM after MI by score. Previous research on whether to impute on item or score-level mostly focused on missing data in the 3-level version of the EQ-5D (EQ-5D-3L), so findings contradicting our findings must be interpreted with caution. Similar to our simulation results, Simons et al. found both MI strategies to be equally accurate for larger datasets (*n* > 500) at all proportions of missing data (up to 40%) and for medium-sized datasets (*n* = 100–500) with mostly UNR (90%) and limited amount of missing data (5–10%). At proportions of 20%-40% missing data (*n* = 100–500), MI at the score-level was found to be more accurate. At sample sizes of n < 100, MI at item-level experienced convergence problems and score imputation was more accurate [1, 2]. This is partially in line with our findings, as the GR CC dataset included 450 patients (falling into range of *n* = 100–500) and we simulated mostly UNR. In accordance with Simons et al. MM after MI at item-level produced similar estimates for 5–10% missing data, whereas for higher proportions of missingness (> 45%) MM after MI at index score-level produced the more reliable results. MI at item-level performed better with increasing INR, especially at higher percentages of missing data [2]. However, in EQ-5D-5L data INR is usually low [42].

In the ABCD dataset results of MM after MI by score and MM after MI by item contradicted the results of Simons et al. despite our larger sample size (*n* = 2040) and a UNR dominated missingness pattern, which highlights the necessity to use “real” observed missing patterns in addition to simulations when investigating missing data analysis. A possible explanation for these results may be that the preference-based scoring system of the EQ-5D can result in larger deviations of the index score when small (single category) errors are made in item-level, thereby limiting the accuracy of the MI by items (Table 2). A level sum score (LSS) for the EQ-5D-5L has been found to be valid and may behave differently than scores generated using utility weights in terms of approaches to missing data. The question of if missing data approaches for the LSS versus utility weighted EQ-5D-5L scores differ should be further explored [43].

This study has several limitations. First, we did not simulate missingness in baseline covariates which is not realistic in real-word data and could lead to different results if covariate missingness is substantial. Secondly, our findings are limited to the MI algorithm using the joint multivariate approach (JM), which is based on the rather strong assumption that the joint posterior distribution of incomplete variables follows JM normal distribution. However, evidence in the literature indicates that JM performs as well as the fully conditional specification (FCS) approach (also called multivariate imputation by chained equations (MICE)), even in the presence of binary and ordinal variables [44, 45]. Moreover, we experienced convergence problems or incomplete imputations at item-level using FCS, which is computationally more intensive per iteration [2, 46]. Future research is needed applying different MI techniques to a range of different sample sizes and scenarios of missingness in time-independent and time-dependent covariates.

The main strength of this study was the parallel analyses of two empirical longitudinal datasets exploring both observed and simulated missing patterns. This approach allowed us to incorporate complex yet realistic associations, meaning that the findings reflect what could be expected in settings with similar patterns of missing data. The simulation analysis (GR dataset) allowed us to determine the accuracy and validate the missing data approaches, while we were able to verify consistency in our findings using the same approaches on “real” observed missing data patterns (ABCD dataset). It is reassuring that overall, the results of simulation and observed data analysis were consistent, although results from the ABCD data did point to complications with baseline covariate missingness which must be further investigated. Furthermore, previous research has not simultaneously addressed the two questions (1) whether to employ MM with or without MI, and (2) whether to deploy MI at item- or at score-level. We comprehensively covered both questions in our study. To the best of our knowledge this is also the first study to give guidance on handling missing data for the 5-level version of the EQ-5D, which is the newer version of the EQ-5D.

## Conclusion

We found that CC analyses overestimated EQ-5D-5L scores and MM after MI by items yielded the lowest scores. As there was no loss of accuracy, MM without MI, when baseline covariates are complete, may be the most parsimonious choice to deal with missing data. Following the principle of parsimony, we would thus recommend applying the simpler approach of MM without MI to handle missingness in the EQ-5D-5L. However, MI may be needed when baseline covariates are missing.

## Supplementary Information

Below is the link to the electronic supplementary material.

Supplementary file1 (DOCX 831 kb)
