# Supplementary material for: What difference does multiple imputation make in longitudinal modeling of EQ-5D-5L data? Empirical analyses of simulated and observed missing data patterns
Source: Qual Life Res. 2021 Nov 19;31(5):1521–32. doi: 10.1007/s11136-021-03037-3 (PMC9023409; doi:10.1007/s11136-021-03037-3)
Supplement: Supplementary file 1 — Supplementary file1 (DOCX 831 kb) [file 11136_2021_3037_MOESM1_ESM.docx]

**Supplements**

**Supplement Table 1:** Baseline characteristics of the ABCD dataset (n=2,040) grouped by completers and patients with missing values.

| **Variable** | **n** | **Full sample**  **(n=2,040)** | **n** | **Complete cases across three evaluated periods (n=1,217)** | **n** | **Patients with missing values (n=823)*** | **p-value** |
| --- | --- | --- | --- | --- | --- | --- | --- |
| Age mean (SD) | 1,985 | 63.07 (13.41) | 1,212 | 63.40 (12.98) | 773 | 62.53 (13.90) | 0.160 |
| Gender  Male n (%)  Female n (%) | 2,027 | 1,110 (54.76%)  917 (45.24%) | 1,216 | 649 (53.37%)  567 (46.63%) | 811 | 461 (56.84%)  350 (43.16%) | 0.120 |
| Marital status  Never married n (%)  Now married n (%)  Separated n (%)  Widowed n (%) | 2,012 | 1,459 (72.52%)  127 (6.31%)  230 (11.43%)  196 (9.74%) | 1,203 | 901 (74.90%)  71 (5.90%)  125 (10.39%)  106 (8.81%) | 809 | 558 (68.97%)  56 (6.92%)  105 (12.98%)  90 (11.12%) | 0.040 |
| Academic status  No formal schooling n (%)  Completed school (1-9) n (%)  High school n (%)  College/University n (%) | 2,028 | 11 (0.54%)  265 (13.07%)  813 (40.09%)  939 (46.3%) | 1,216 | 3 (0.25%)  141 (11.60%)  473 (38.90%)  599 (49.26%) | 812 | 8 (0.98%)  124 (15.27%)  340 (41.87%)  340 (41.87%) | 0.002*  <0.001** |
| Single self-rated health (SRH)  Excellent  Very Good  Good  Fair  Poor | 2,004 | 71 (3.5%)  611 (30.5%)  926 (46.2%)  331 (16.5%)  65 (3.2%) | 1,200 | 48 (3.9%)  387 (32.3%)  546 (45.5%)  193 (16.1%)  26 (2.2%) | 804 | 23 (2.9%)  224 (27.9%)  380 (47.3%)  138 (17.2%)  39 (4.9%) | 0.003§  <0.001** |
| EQ-5D-5L index first evaluation mean (SD) | 2,019 | 0.795 (0.169) | 1,217 | 0.809 (0,157) | 802 | 0.774 (0.184) |  |
| EQ-5D-5L index second evaluation mean (SD) | 1,507 | 0.793 (0,168) | 1,217 | 0.799 (0,166) | 290 | 0.768 (0.176) |  |
| EQ-5D-5L index third evaluation mean (SD) | 1,374 | 0.788 (0,173) | 1,217 | 0.792 (±0,169) | 157 | 0.758 (0.198) |  |

*Missing values in at least one of the three evaluated periods. ^§^Chi square or Fisher test. **Mantel-Haenszel test of linear association

**Supplement Table 2:** Mean EQ-5D-5L index scores at baseline and follow-up (ABCD dataset) and predicted values for the four approaches

|  | n | Baseline | 1 year | 2 years |
| --- | --- | --- | --- | --- |
| EQ-5D-5L index scores total cohort mean (SE) | 2,036 | 0.796 (0.004) | 0.793 (0.004) | 0.788 (0.005) |
| EQ-5D-5L index scores complete cases mean (SE) | 1,217 | 0.809 (0.005) | 0.799 (0.005) | 0.792 (0.005) |
| EQ-5D-5L index scores MM complete cases* mean (SE) | 1,217 | 0.809 (0.002) | 0.803 (0.003) | 0.793 (0.003) |
| EQ-5D-5L index scores MM total cohort* mean (SE) | 2,040 | 0.796 (0.002) | 0.792 (0.002) | 0.786 (0.002) |
| EQ-5D-5L index scores MI by score and MM*^§^ mean (SE) | 2,040 | 0.796 (0.001) | 0.794 (0.002) | 0.790 (0.002) |
| EQ-5D-5L index scores MI by items and MM*^§^ mean (SE) | 2,040 | 0.794 (0.001) | 0.786 (0.002) | 0.776 (0.002) |

MM: Mixed Model; MI: Multiple imputation; SE: standard error; *predicted values **^§^**pooled by Rubin´s Rules.

**Supplement Table 3:** Pooled mean and standard error of EQ-5D-5L predicted values by each approach (MAR), GR dataset

|  | **Baseline (T0)** | | **Post-treatment (T1)** | |
| --- | --- | --- | --- | --- |
|  | Mean | SE | Mean | SE |
| *Original dataset* | *0.7055* | *0.0055* | *0.7615* | *0.0059* |
| **MAR 5%** |  |  |  |  |
| MM complete cases | 0.7105 | 0.0055 | 0.7670 | 0.0058 |
| MM without MI | 0.7059 | 0.0054 | 0.7624 | 0.0058 |
| MM after MI by score | 0.7057 | 0.0055 | 0.7622 | 0.0059 |
| MM after MI by item | 0.7043 | 0.0056 | 0.7601 | 0.0060 |
| **MAR 10%** |  |  |  |  |
| MM complete cases | 0.7146 | 0.0055 | 0.7713 | 0.0058 |
| MM without MI | 0.7060 | 0.0054 | 0.7630 | 0.0058 |
| MM after MI by score | 0.7054 | 0.0056 | 0.7624 | 0.0060 |
| MM after MI by item | 0.7030 | 0.0057 | 0.7588 | 0.0061 |
| **MAR 15%** |  |  |  |  |
| MM complete cases | 0.7180 | 0.0055 | 0.7751 | 0.0059 |
| MM without MI | 0.7057 | 0.0054 | 0.7629 | 0.0058 |
| MM after MI by score | 0.7054 | 0.0057 | 0.7617 | 0.0061 |
| MM after MI by item | 0.7046 | 0.0057 | 0.7570 | 0.0061 |
| **MAR 25%** |  |  |  |  |
| MM complete cases | 0.7228 | 0.0057 | 0.7801 | 0.0061 |
| MM without MI | 0.7055 | 0.0055 | 0.7631 | 0.0058 |
| MM after MI by score | 0.7054 | 0.0058 | 0.7616 | 0.0062 |
| MM after MI by item | 0.7038 | 0.0059 | 0.7515 | 0.0063 |
| **MAR 35%** |  |  |  |  |
| MM complete cases | 0.7293 | 0.0060 | 0.7865 | 0.0064 |
| MM without MI | 0.7053 | 0.0055 | 0.7635 | 0.0059 |
| MM after MI by score | 0.7052 | 0.0059 | 0.7606 | 0.0063 |
| MM after MI by item | 0.7038 | 0.0061 | 0.7451 | 0.0064 |
| **MAR 45%** |  |  |  |  |
| MM complete cases | 0.7355 | 0.0062 | 0.7940 | 0.0066 |
| MM without MI | 0.7054 | 0.0055 | 0.7660 | 0.0059 |
| MM after MI by score | 0.7052 | 0.0060 | 0.7635 | 0.0064 |
| MM after MI by item | 0.7039 | 0.0061 | 0.7419 | 0.0065 |
| **MAR 55%** |  |  |  |  |
| MM complete cases | 0.7402 | 0.0067 | 0.7989 | 0.0072 |
| MM without MI | 0.7051 | 0.0056 | 0.7657 | 0.0060 |
| MM after MI by score | 0.7050 | 0.0061 | 0.7620 | 0.0065 |
| MM after MI by item | 0.7037 | 0.0061 | 0.7350 | 0.0065 |
| **MAR 65%** |  |  |  |  |
| MM complete cases | 0.7427 | 0.0076 | 0.8015 | 0.0082 |
| MM without MI | 0.7053 | 0.0057 | 0.7644 | 0.0061 |
| MM after MI by score | 0.7052 | 0.0061 | 0.7594 | 0.0066 |
| MM after MI by item | 0.7039 | 0.0061 | 0.7278 | 0.0065 |

MAR: Missing at random; MM: Mixed model; MI: Multiple imputation, SE: standard error

**Supplementary Figure 1:** Pattern of simulated data at different percentages of missing data (MAR), GR dataset (n=450)


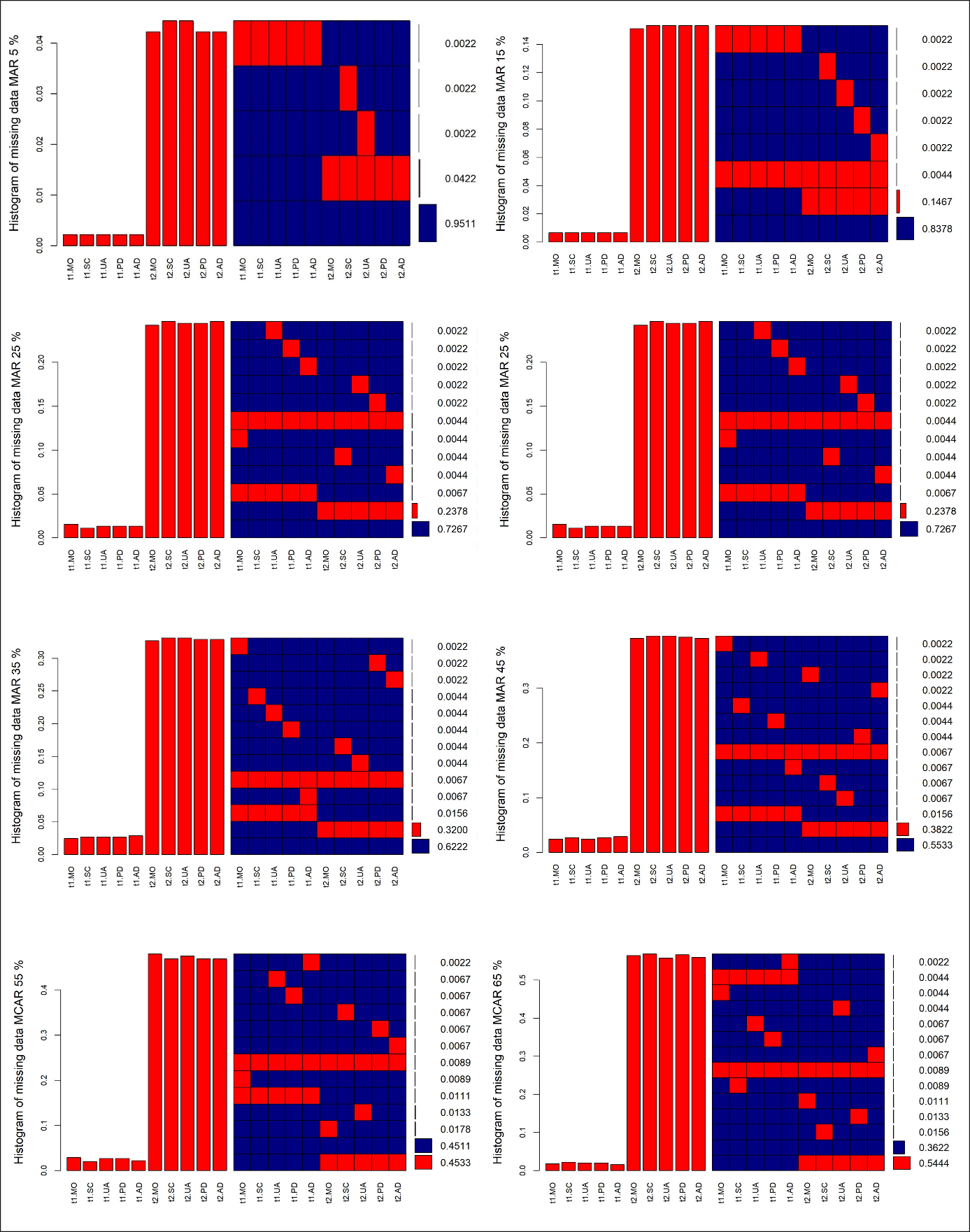


MO: mobility, SC: self-care, UA: usual activities, PD: pain/discomfort and AD: anxiety/depression

**Supplement Table 4:** Pooled beta coefficients of the fixed effect time by each approach (MAR) for GR and ABCD dataset and original dataset

| GR dataset | | | | |
| --- | --- | --- | --- | --- |
| **Complete dataset (n=450)** | **β*time_GR* = 0.026** | | | |
|  | **Approach 1** | **Approach 2** | **Approach 3** | **Approach 4** |
| **β*time*** | **MM only complete cases** | **MM total cohort** | **MM after MI by score** | **MM after MI by items** |
| **MAR 5%** | 0.027 | 0.027 | 0.026 | 0.025 |
| **MAR 10%** | 0.028 | 0.027 | 0.026 | 0.025 |
| **MAR 15%** | 0.028 | 0.028 | 0.025 | 0.021 |
| **MAR 25%** | 0.029 | 0.028 | 0.024 | 0.015 |
| **MAR 35%** | 0.031 | 0.028 | 0.022 | 0.008 |
| **MAR 45%** | 0.034 | 0.030 | 0.025 | 0.004 |
| **MAR 55%** | 0.034 | 0.030 | 0.023 | -0.003 |
| **MAR 65%** | 0.034 | 0.028 | 0.020 | -0.010 |
| ABCD dataset | | | | |
| **β*time*** | -0.002 | -0.004 | -0.002 | -0.008 |

MAR: Missing at random; MM: Mixed model; MI: Multiple imputation
